# Women’s Experiences of Domestic Violence during Pregnancy: A Qualitative Research in Greece

**DOI:** 10.3390/ijerph17197069

**Published:** 2020-09-27

**Authors:** Evangelia Antoniou

**Affiliations:** Department of Midwifery, University of West Attica, 12243 Psachna, Greece; lilanton@uniwa.gr; Tel.: +30-693-720-5679

**Keywords:** violence, pregnancy, partner, risk factors, nationality

## Abstract

This qualitative research is the second part of a quantitative research that aims at recording the phenomenon of violence in pregnancy. The first part was carried out during August and September 2009 (N = 546). It was found out that the rate partner’s violence was 6%, while for 3.4% of the pregnant women, abuse started after the pregnancy. In the second part of this research, the semi-structured interview was used to investigate the way pregnant women experience violence. The sample comprised seven women abused by their partner (Ν = 7) at the women’s shelters of “Mitera” Babies’ Center and the National Social Solidarity Center between September 2010 and December 2011 and who accepted to participate in the research. The targets of the research were the investigation of the risk factors for the manifestation of violence, the profile of the victim and the perpetrator, the consequences of abuse for the woman, her reproductive health and the fetus. The majority of the abused pregnant women were foreigners and only two were Greek. The latter had experienced severe traumas (physical and psychological) since their childhood. Violence in their lives is the main characteristic of the foreign women seeking a better life in Greece, too. Alcohol use or abuse by the partners, poor socioeconomic background of the mothers and their partners, and pregnancy per se are the main risk factors of the violence against women in this period of their lives. Violence resulted in miscarriage in one case, while abortion was the alternative chosen by another as a solution to social exclusion and possible domestic violence. Anxiety and despair were the main psychological consequences. The small number of women included in the collection of qualitative data is a limitation for the research and decreases the reliability index of its results.

## 1. Introduction

According to UN declaration, domestic violence is “the violence occurring in the family or domestic unit, including, inter alia, physical and mental aggression, emotional and psychological abuse, rape and sexual abuse, incest, rape between spouses, regular or occasional partners and cohabitants, crimes committed in the name of honour, female genital and sexual mutilation and other traditional practices harmful to women, such as forced marriages” [1].

A survey [2] was carried out in 2003 with the aim to record the nature and magnitude of domestic violence in Greece. In a sample of 1200 women, aged 18–60, 61.9% of women living in semi-urban centers stated that they frequently experience verbal or psychological abuse by their husband/partner. The percentage was slightly decreased for women living in urban centers and agricultural areas. Furthermore, 5.3% of the women in semi-urban centers, 3.7% in urban and 3% in agricultural areas stated that they have been victims of physical violence. Finally, 3.3–4% of women, depending on the area of living, stated that they are coerced to have sexual intercourse. These results constituted the leverage for political, social and legislative measures to deal with violence against women in the family [3]. In a recent review of 92 studies held in 23 countries, the average rate of physical violence in pregnancy was 13.8%, that of sexual abuse was 8.0% and that of emotional abuse was 28.4% [4].

### 1.1. Domestic Violence in Pregnancy

Domestic violence in pregnancy is a serious public health problem that can put the life of the pregnant woman and the fetus at risk [5,6]. The incidence of the phenomenon varies depending on the definition, the context, the material and the methodology used in investigating violence [4,7,8].

According to the study of Tailleu and Brownridge [9] on the prevalence of violence in pregnancy, the landmark was the publication of an article by Gazmararian et al. in 1996 [10], reviewing all studies up to date on the phenomenon of violence in pregnancy. This article found that in developed countries, the percentages of violence in pregnancy ranged to a great extent from 0.9% to 20.6%, whereas the majority ranged between 3.09% and 8.3% [11,12,13,14,15,16,17,18,19,20].

The surveys that followed concerned both the developed and the less developed countries and the various types of violence (physical, sexual, emotional and verbal). It was shown that higher percentages of prevalence of the phenomenon are linked to younger age, lower income and unmarried women [21,22,23]. The measurements of violence including multiple data and with reference to different types of violence tend to result in higher percentages of the prevalence of the phenomenon [24,25].

The cultural differences between developed and non-developed countries in matters of violence may account for the differences in percentages in relation to violence before and during pregnancy. An intercultural survey showed that the degree of imbalance of the two genders in a society affects the percentages of violence in the general population of women [26] and may also be a factor for violence in pregnancy.

With regard to the victim’s characteristics, the key risk factors that seem to increase the prevalence of violence in pregnancy are a history of violence in a woman’s life [19,22,27,28] followed by origin, with higher percentages of victims among women belonging to minority groups [29,30,31], particularly a low educational level in combination with a low socioeconomic level [24,29,30], substance abuse [29,31,32,33,34,35,36,37] and, finally, adolescence [17,38,39].

In terms of the key characteristics of the perpetrator as for risk factors increasing the incidence of violence in pregnancy, exercise of power and control [40,41] and substance abuse [19,20] are the main reasons, as shown in relevant studies, for violence by women’s partners.

A third group of factors linked to the increase in violence in pregnancy is related to the circumstances and conditions of pregnancy. Undesirable pregnancy and accidental or unplanned pregnancy constitute the most important factors of violence in pregnancy [19,42,43].

The exercise of violence in pregnancy has disastrous consequences both for pregnant women and fetuses. More precisely, the consequences observed concern women’s mental health [44,45]. Women victims of domestic violence are isolated and usually suffer from depression, eating disorders, panic attacks and anxiety. They also harm themselves and the fetus with alcohol, drug abuse or medicines [35,36,46]. Other consequences concern obstetric and gynecological complications (recurrent miscarriages, hemorrhage, premature rupture of membranes, premature birth, premature placental abruption) [22,44,45,47,48]. Last but not least, complications may affect the fetus too (low birth weight) [17,49,50,51]. International surveys have suggested that women who were victims of family violence used healthcare services less and delayed prenatal care [52,53]. Midwives and any health professionals responsible for the care of pregnant women should cooperate with them and try to understand if the reason for certain behaviors (i.e., psychological problems, drug use and substance abuse) is related to cases of domestic violence.

### 1.2. Domestic Violence in Pregnancy in Greece

The disconcerting results of the 2003 Greek study and the studies from other countries on domestic violence in pregnancy made the investigation of domestic violence in pregnant women in Greece necessary and urgent, although it is possible that the phenomenon was not accurately recorded due to the refusal of the victims to voluntarily reveal their silent acceptance of violence.

The statistical research [3] held from August to September 2009 by the author of this article constitutes the quantitative-statistical part of the first research ever carried out in Greece on domestic violence in pregnancy. This publication of the researcher deals with the second part of the qualitative recording of the phenomenon. The participating pregnant women in this statistical research comprised 546 women who agreed to answer the questionnaire given to them during their visit to the outpatients’ clinics of the Regional General Public Hospitals “Alexandra” (Ν = 264) and “Elena Venizelou” (Ν = 282) of Athens. The questionnaire was translated into the Greek language and weighted by the Abuse Assessment Screen (AAS) researcher-midwife.

The results of the quantitative research show that 6% of the pregnant women are abused by their partners; this percentage falls within the range (0.9–22.0%) given by corresponding studies in other European and North American countries.

The percentage of the phenomenon was detected as being slightly over the lowest limits of the corresponding percentage in the general women’s population, as shown by the first epidemiological study on domestic violence against women in Greece (3–5.3%). More specifically, 23% of the sample, 119 out of 546 pregnant women, stated that they had suffered emotional or physical pain. Physical pain was reported by 8.6% of the sample; slapping or pushing was the most frequent type of violence for 2.7% of the abused pregnant women and as such, this type of violence is not stigmatized or considered an injury of a specific part of the body. Emotional pain was reported by 22.6%. The onset of pregnancy seemed to be a protective factor against physical violence for some women; however, physical violence still remained at high levels, even in this vulnerable period of a woman’s life: 2/3 of the abused women (3.4% of the sample vs. 5% stating that they were abused the previous year) stated that they still experienced violence even after the onset of pregnancy, without being clarified by the quantitative research if there were cases that violence started during pregnancy.

The aim of this research is to study in depth as much as possible the experiences of pregnant women who are abused and highlight the factors associated with the abuse and the consequences for women and fetuses.

## 2. Materials and Methods

### 2.1. Selecting Qualitative Research

According to a standard definition, qualitative research is an interpretive study of a specified issue or problem in which the researcher is central to the sense that is made [54]. The word qualitative (research) underlines the emphasis on the quality of the entities under study and the processes and meanings that cannot be studied experimentally or be measured in relation to the quantity, degree, intensity or frequency.

Qualitative methodology focuses on the meaning—that is, how people understand and make sense of the world, experience and themselves. For the collection and analysis of the data of this research, the interpretative phenomenological analysis was chosen.

Phenomenology can be defined as: “the study of phenomena (i.e., the structures of consciousness) as experienced by our consciousness, without theories regarding their causal explanation and with as much freedom as possible from prejudices that have not been examined, and from preexisting assumptions” [55].

The aim of a phenomenological study is to produce a full description, as much as possible, of the phenomena of daily experience with the ultimate aim to separate the aspects of the phenomena that are related to specific circumstances from their essence, which is considered stable and unchanged. In other words, the distinction between the phenomenon that can vary more or less and the rationale that governs it, which is its unchanged essence, is imperative. In this research, we tried to show the essence of violence in pregnancy, its unaltered characteristics and distinguish them from specific incidents.

The phenomenological method to acquire understanding includes three distinct phases: (1) epoch, (2) phenomenological reduction and (3) imaginative variation [55]

1. Epoch—originating from ancient Greek with the meaning of withholding of assent—requires the suspension of preexisting assumptions, judgments and interpretations of the researcher about the phenomenon to be allowed to acquire full knowledge of the phenomenon.
2. The second phase is transcendental—phenomenological reduction. In this phase, it is not only the description but also the personal experience that plays a role. This means that an assumption about the real essence of the object is put in brackets and the researcher focuses on consciousness. The main interest in this research is to highlight what the woman has perceived as experience, as characteristic of the violence inflicted on her and as a realizing incident as she can consciously express it today.

The aim is to describe what we say not only in terms of an external object, in this case the phenomenon of violence, but also describing the internal act of consciousness, its mental consequences, the experience per se, the rate and the relation between the phenomenon and the person, using a language that exposes the “feeling” of the experience. In other words, we did not just record the incidents or episodes but also tried to highlight the qualitative characteristics of the experience with the aim of bringing to light, through the personal narration, the emotional, mental trait that experience has left. The steps comprising the phenomenological reduction include:

(a) Bracketing the focusing point of the research. We do not focus on violence per se from the beginning to the end.
(b) Horizonalization, which means that each sentence is initially treated as having the same value with all the others and only later the thematic topics, the “noema horizons”, are distinguished. In our analysis, therefore, we initially evaluated each sentence of the interviewed woman.
(c) The horizons are grouped in topics, and the thematic topics that are set out below.
(d) Finally, we organized the horizons, the points of view according to which violence shall be studied and also, the thematic axes, in a comprehensive description of the phenomenon.

3 The imaginative variation follows the transcendental-phenomenological reduction phase and aims at providing the structural substance of the experience, to create, that is, a picture of the required conditions for an experience to occur. In this context, we shed light on the background for the violent episode, what has preceded, what followed, the feeling that dominated etc.

The aim is to reach a structural description of the experience, the factors explaining what is experienced; in other words, the “how”, which refers to the conditions illuminating the “what” of the experience. In the imaginative variation, we leave the world of the things and measurable entities to go towards “noema” (meaning) and substance. Free imagination is combined and interchanged with thinking about processing the possibilities. For example, we moved from the development of the episode to the experience during the violence, but also before and after.

### 2.2. Observing Bio-Ethic Rules

Permission was given by the Administrative Board and the scientific competent person of “Mitera”, Babies’ Center, and the Administration of the National Social Solidarity Center (EKKA) for the interviews in the framework of the qualitative research. Both these institutes are in Attica and provide protection and support to single-parent families with serious social, psychological or financial problems, adolescent mothers, pregnant women who are alone, abused mothers and abused, neglected or abandoned children. A necessary condition for the interview was the provision of information about the research and the signed consent of the pregnant women. The consent form included the commitment of the researcher to keep the confidentiality of personal data and another form the commitment of the researcher not to disclose the place where the women were accommodated. Support services of the reception centers were at the disposal of the respondents after meeting the researcher if the intervention of the mental health experts, legal persons etc. was deemed necessary.

### 2.3. Selection and Limitations of the Sample

The sample of the research comprised 7 women (Ν = 7) who found shelter at the women’s shelters of “Mitera” Babies’ Center and the National Social Solidarity Center because of their partner’s abuse in the period September 2010–December 2011 and who accepted to participate in the research. The next chapter shall make a detailed reference to the selection of the sample.

It is usual in qualitative research to determine the number of interviews from the degree of difficulty in accessing the respondents. The difficulty of investigating violence in pregnant women using the interview as a tool is unquestionably a significant factor that is often determined by the refusal of the existing institutes to give permission. It is almost a necessity due to the used data collection method and the data analysis procedures that de facto restrict the number of people used to collect the data.

In Greece, hostels and, in general, the bodies providing help and protection to pregnant women are few and unknown to the public. On the other hand, there is a stigma and taboo regarding finding shelter for the woman; as a result, she turns to this type of accommodation only when all the other possible environments have turned her down. As long as the family is a key protective social web of Greek society, the number of victims of incidents of violence that shall seek shelter will remain low. However, even when seeking shelter, as the role of the bodies is to protect them from any further injury that a memory of the traumatic incidents might inflict, it is difficult for the researchers to approach them. As many times there are serious issues for the future of the pregnant woman and her child to be born at stake (i.e., adoption) which make the presence of the researcher awkward, discretion and fine communication manipulations by the researcher are in any case necessary so that the intervention is as painless as possible.

### 2.4. Planning and Research Setting

This qualitative research is the second part of a research project that aims at recording the phenomenon of violence in pregnancy. The first part was carried out in the period August–September 2009 in a sample of 546 women. Pregnant women followed up during their pregnancy by the 2 public maternity hospitals (Alexandra and Elena Venizelou) in Attica took part in the study. It was found out that the rate of partner’s violence is 6%, while for 3.4% of the pregnant women, abuse started after the pregnancy. In addition, the quantitative research showed that nationality, of both the victim and the perpetrator, is associated to a significant degree with violence during pregnancy, along with the history of miscarriages and undesired pregnancy.

In the second part of this research, the semi-structured interview was used to investigate the way the experience of violence is recorded by the pregnant women, using the phenomenological approach. The researcher called the directors of the hostels on a weekly basis, who informed her about the possibility of a voluntary participation in the research of a pregnant woman. All pregnant women that accepted to take part were aware that they were participating in research on violence in pregnancy and agreed to be recorded, provided secrecy was kept.

### 2.5. Data Collection

The interview took place at an office provided at the two hostels of EKKA and at a hostel of “Mitera”. During the interview, the researcher was alone with the pregnant woman, with the exception of a pregnant woman with a history of rapes by persons in her family, in which case it was deemed necessary to have a social worker, trusted by the pregnant woman, present.

### 2.6. Interview as a Methodological Tool

The interview as a methodological tool has some advantages that make it a useful tool which, along with the structured questionnaire, provide a comprehensive picture for the results of our research. First of all, the interview allows a research problem to be studied in depth. Once the researcher manages to establish a comfortable and friendly relationship with the other person, it is quite possible to obtain confidential and personal information that the respondent would be unwilling to be recorded. This is why the researcher has to keep a bracketing attitude, which means empathy, acceptance, understanding, and honesty, to transfer to the respondent exactly what he/she feels or thinks, keep his/her distance from subjective views and show a high feeling of responsibility.

Moreover, another key advantage of this tool is that through the interview, the researcher can lead the person to a higher level of soul-searching and investigate important aspects of the abusive behavior, in this case.

Finally, through the interview, the researcher can more easily explain the purpose of the research, keep the interest and be sure each time that he/she fully understands the question and that the necessary information is given.

In the specific research, the semi-structured interview was selected for the procedure that gives significant freedom to the researcher to change the procedure, if necessary, should there be resistance or necessary supplementary questions. An interview guide with six thematic categories corresponding to the six targets of the research was created. This paper shall present the findings of the following two thematic categories:

- The thematic categories corresponding to first target of the research, i.e., (1) the risk factors for the manifestation of violence, (2) the character of the victim and (3) the perpetrator, were:
  - Victim’s profile
  - Description of incidence of violence (circumstances, description of experience)
  - Perpetrator’s profile before pregnancy
  - Description of the dynamics of the relation with the partner (before pregnancy)
  - Interpretation for hypothetical causes of violence
- The thematic categories corresponding to second target of the research, i.e., (1) the consequences of abuse for the woman, (2) her reproductive health and (3) the fetus health, were:
  - Impact of violence on the woman’s physical and reproductive health
  - Impact of violence on the fetus’ physical health.

Presentation of the findings and interpretation of the content of the thematic categories. Support of the findings by presenting parts of the woman’s responses and, finally, commenting on the findings based on other theories and literature references.

The questions raised in the semi-structured interview corresponding to the two thematic categories were:

(A) Investigation of factors initiating the abuse
  - When did you notice the first signs of violence by your partner (abusive—reviling speech, shouting, pushing, hitting, destruction of objects, forcing sexual intercourse etc.)?
  - Has it crossed your mind to terminate your relation after the first signs of violence?
  - Why didn’t you do it?
  - Can you describe a recurrent incident of violence?
  - What were the circumstances? For you? For him?
  - How did you treat your partner after his violent outbreaks?
  - Did you have any problems then?
  - Was it different during pregnancy? New episodes?
  - How do you explain your partner’s violent behavior?
  - How does your partner explain his violent behavior?
  - How did you react in the first violent episode?
(B) Investigating the consequences of domestic violence on the quality of reproductive health
  - How many times have you been pregnant?
  - How many children do you have? Were those planned—desirable pregnancies or accidental?
  - How many miscarriages? Why? Was there an incident of violence? At what pregnancy age?
  - How many abortions? Why? Was there an incident of violence? At what pregnancy age?
  - Have you undergone artificial insemination?
  - Have you attended pregnancy preparation classes?
  - Have you done all the tests? Are you monthly followed up?
  - Did you have health problems during pregnancy because of violence (contractions, hemorrhage, vomiting)?
  - How have the incidents of violence affected your sleep, appetite, stress events during pregnancy (crying, intense sadness, irritability etc.)?

### 2.7. Data Analysis Procedure

After the recording of each interview, the researcher recorded the key axes (thematic) of the problems of each abused pregnant woman. In the week that followed the interview, she wrote the script of the interview and then decoded the material (initiation of violence, type of violence, problems in pregnancy, perpetrator’s profile etc.). Then, the topics were aligned with the thematic/research objectives. Finally, the words or phrases of an interpretative nature were included with subjective justification of the facts.

## 3. Results

### 3.1. Profile o Fpregnant Woman

For the data collection and analysis of this research, interpretative phenomenological analysis was selected. We described the phenomenon in its entirety in the phenomenological reduction. We tried to have a complete picture, as much as possible, of the range of factors associated with the violence inflicted by the partners to the pregnant women of the sample, stressing aspects of possible causes and consequences and the personality of perpetrators and victims.

The thematic sessions were decided based on the investigation of specific factors relating to the abuse start, its consequences in terms of the partner, children and, in general, the family, its consequences on the quality of reproductive health and the woman’s professional and social life, the degree of using health and welfare services and, finally, the pregnant woman’s ideas about the role of the genders and ways to deal with violence.

Four (4) out of the seven (7) women who participated in the survey were living at the Guesthouse for abused women of the National Social Solidarity Center and three (3) at the “Mitera” Infant Centre. Five (5) were foreign and two (2) were Greek. The key characteristics of the two Greek women was their young age (17 and 22 years old) and the major social (isolation from the social web, extremely low educational level) and psychological problems manifested due to the abuse by members of the family. It is worth noting that both live in remote areas, away from urban centers, and are not married or have ever lived with a sexual partner (Table 1).

These are cases that the abuse—repeated sexual abuse in one case and physical, verbal, psychological and sexual harassment in the other—does not come from the partner but from the father, mother, siblings or grandfather. In the first case, there were pregnancies from rapes by incestuous family members, resulting in the adoption of the babies (Table 2).

We could say that in terms of the Greek population, only especially problematic, social-welfare cases resort to guesthouses for abused women. This fact suggests that the less socially vulnerable groups of abused women either do not speak about their problem as they are afraid to be stigmatized or they seek help from experts on a private basis or are supported by family and friends.

The characteristic of the abused pregnant women from the Balkans (Albania, Romania), Eastern European countries (Moldavia, Georgia) or African countries (Morocco) is the lack of security in their social, work and financial conditions governing their lives in Greece. In most cases, they are unemployed, having entered the country illegally, they face serious inclusion problems and, sometimes, fall victims of exploitation by their partners. Although their educational level ranges from fundamental to good (one case mentioned that she had studied pedagogics), they are involved in abusive relationships (Table 1). It is important to add that all pregnant women participating in the sample had either suffered violence by family members or were witnesses of the father’s violence towards the mother. Furthermore, in two cases, pregnant women reported violence both by their ex-husband in their homeland as well as by their current partner whom they met in Greece (Table 2).

### 3.2. Perpetrator’s Profile

The perpetrator in five (5) out of the seven (7) cases is the sexual partner or husband. We do not have sufficient data about the partners of the abused pregnant women. In general, he is of the same age, foreign, of common origin with the pregnant woman and with a lack of security for his work and financial condition. In most of the cases, he is unemployed and, quite frequently, his objective is to financially exploit the pregnant woman. Only in one case the current—second—husband is Greek, much older than the pregnant woman (by about 30 years) and works as a free-lancer (taxi driver) (Table 3). The women presented the men as immature and insecure personalities, with a predisposition for alcohol, other illegal substance abuse and illegal activities (gambling, prostitution etc.). Jealousy and insecurity are the main characteristics driving them to impulsiveness and violence.

*Lena: -He was very jealous. I was at home. […] Stayed at home all the time. And he used to call me 10 times. Where are you; What are you doing; If I was going to the supermarket, where did you go?*

*-What would you say were the reasons for your partner’s violent behaviour? What were they?*

*Marina:-Drugs and financial problems.*

*-Yes, he was problematic. He did not want me to have friends, talk to my mother, go out. Do nothing. Either with friends or anybody else. Whenever I was to go out, I had to go with my child. Never alone.*

*Giota: -He was all the time in jail. More months he was in jail.*

*[…] He used to come home late, he took all my money*

*-But we had to pay the rents; he was not working; he was expecting me to pay. He would spend all the money.*

*[…] my husband used to beat me because he did not have money or drugs or this or that.*

He prostituted me in Omonia square * in the end. I was pregnant.

* Place where prostitutes hang around.

In the two cases of the Greek pregnant women, the perpetrator was a family member.

*Lambrini: -The person that harassed me for the first time and raped me forcing me to have double abortion was my grandfather. My mother’s father.*

*My father also raped me. […] this is why, he is in jail. Not only me. My sister as well. […] I was never pregnant by my father. My father is in jail for statutory rape.*

*-Were you beaten by your sister, your brother?*

*-Not by my sister. By my brother, yes.*

*-One of your brothers?*

*-All of them.*

*Ariadni: -Sometimes when my mother used to shout at me, I pretended that I did not listen and we ended up fighting […]*

*-Have they been beating you for many years?*

*-Since I was a child.*

*-Since you were born?*

*-Yes, yes.*

*-Why do you think your brothers used to beat you?*

*-Because they were drunk.*

*-Look, one of my sisters, L…, is with my brother, M…*

*-As a couple?*

*-Yes.*

*-And what do you say to her?*

*-Not talk back and not cause trouble.*

*-Is P…. your boyfriend?*

*-No, my brother.*

*-What did he want? To be a couple with you?*

*-Yes, but at some point, I said I am going to the prosecutor, but by mum said that I shouldn’t talk.*

*Violence in pregnancy: start, nature of violence, characteristics of pregnancy*

The abuse started in pregnancy in three (3) cases, while violence preexisted in the case of two (2) Greek and two (2) foreign women. In the cases that violence preexisted, it became more frequent in pregnancy (Table 2).

*Marina: -When he found out that I am pregnant, something changed […] He shouts all the time*

*-Was he beating you before you got married?*

*-No.*

*-This started in pregnancy or before?*

*-After we got married, when I became pregnant.*

*-You got married and you were pregnant?*

*-Yes*

In all cases, violence is of a psychological nature. Pregnant women report that with all the threats, insults and shouting by their husbands, they were living in a state of terror and permanent stress. The woman from Moldavia (Table 2) answered our question about the stress:

*-Lena: -Very much. Each day […] This is why, I could not stand anymore […] I said I will leave. I will sleep outdoors […] He said that he wants to help me with the baby, but I do not want to go back. Never. Because I now… again crying, again the same. I do not want. And I cannot forget everything that he has said to me.*

Verbal abuse was daily in some cases.

*-He used to swear. Every night, I was crying, crying, crying. And I thought about the baby. The baby understands everything.*

And was aimed at the older child of a pregnant woman

*-I could not talk balk. He used to swear at my daughter as well.*

Physical abuse was mainly aimed at the face and legs, but rarely at the abdomen. The partner often kicked, pushed, slapped the pregnant woman, sometimes in front of her other child. In one case, he was so violent that he broke his wife’s teeth, and, in another case, he threatened her with a weapon:

*-Giota: -[…] Beat me hard, swearing, swearing in front of the child, saying different things*

*-[…] My face was covered with blood; I went to the hospital and he went home to sleep as if nothing had happened.*

*I told them that he had a weapon, a weapon, or he used to say that he would kill my child.*

*-Did your first husband, from Morocco, beat you every day?*

*-Susanna: -Yes.*

*-And he took all your money.*

*-Yes.*

*-And he broke your teeth.*

*-Yes.*

Apart from the two cases of sexual abuse of the two Greek women by family members, the partners of the pregnant women demanded sexual intercourse and cases of rape or prostitution were reported in pregnancy:

*-Did he force you to have sex?*

*-I do not want to talk about it.*

*Marina: -He started hanging around with some people that he shouldn’t, because one of them was a pimp and other things… and from then on, he changed, he started swearing, beating me…*

*Giota: -He prostituted me in Omonoia square in the end. I was pregnant.*

For the pregnancies, the women’s partners, with the exception of one case, had not said that they were unwanted, although unscheduled:

*-No, because, fortunately, he did not hit me hard. He did not manage to hit hard but he was telling me to get rid of the baby. If you do not go to the doctor to do it, I have another solution, he said. I can do it for you.*

However, the participants justified the abusive behavior of their partners as fear of assuming the responsibility of fatherhood:

*Lena: -[…] As I realized he changed because of the baby.*

*“* *When he heard that I was pregnant, he changed, as if he was afraid.”*

In one case, the husband’s violent behavior agrees with the social morals of the country of origin. In Morocco, the country of origin of one abused woman, women do not enjoy many freedoms or rights and the husband has the absolute control over his wife’s life. Even the right to beat her.

*That’s how things are in Morocco.*

*Susanna: -Yes.*

*The man wants to control the woman.*

*-When he leaves with a friend, he beats me. And I want it.*

*-You want to get beaten?*

*-If I don’t leave.*

*-Yes.*

*Before getting married with M, I used to stay at home…*

### 3.3. Consequence of Violence on the Physical and Reproductive Health of the Pregnant Woman and the Fetus

In general, no consequences are reported on the reproductive health of the pregnant woman or the fetus due to the partner’s violence. In one case of violent abuse in the past, the pregnant woman had to go to the hospital for sutures on the face, but this did not affect her pregnancy, because she was not beaten at the abdominal region but on the face.

*-Did you have any health problems now in this pregnancy or in the previous one due to violence?*

*Lamprini: -No, no.*

*-Bleeding…*

*-Nothing…*

*-Did your first husband, from Morocco, beat you every day?*

*Susanna: -Yes.*

*-And he took all your money.*

*-Yes.*

*-And he broke your teeth.*

*-Yes. And he stays with the children and I go to work.*

*-Yes*

*-And he takes all my money.*

Furthermore, two foreign pregnant women reported that in the past, they had a miscarriage in a pregnancy from an ex-partner or an ex-husband due to abuse and a Greek woman had a double abortion because she did not want to give birth to children resulting from incest (Table 1).

## 4. Discussion

The aim of this qualitative research was, as mentioned above, the investigation and in-depth study of the abuse of women by their partners during pregnancy. This is why it was deemed necessary to focus on the start of violence, the profiles of both the pregnant women and the perpetrator and the dynamics of their relationship as it was before and after pregnancy. The seven pregnant women were invited to give a possible interpretation of the violent behavior of their partner or the family member and also describe the consequences of the abuse on their mental and physical health and the physical health of the fetus.

The first finding worth commenting is about the characteristics of the sample: there were only seven pregnant women, out of whom only two Greek women, in a period of 1 year that sought help from the two most well-known shelters for abused women in Attica. Before studying the profile of the foreign abused pregnant women, we shall focus on the particularly traumatic characteristics of the two Greek women. They were two young girls living away from urban centers and violence was a daily experience since they were born either as witnesses of the abuse of others or as victims. Violence was for them a normality in their lives. The first one was beaten daily by her brothers and mother without any reason and suffered sexual harassment by her brother. Incest was almost something normal in the family, as her sister had a sexual relationship with her brother. Her pregnancy, which was realized after the fourth month, stopped the abuse behavior by the other family members, especially as there was a welfare representative observing the family, but it was seen as an undesired condition that most probably would result in giving the baby up for adoption. The second case is even more representative of the traumatic family history that abuse in pregnancy may hide. It was not the first time that the 17-year-old girl was raped by a member of her family and became pregnant. In the past, she had had two abortions, one for twins, as she was raped by her maternal grandfather. The father’s filing a complaint led to the grandfather’s imprisonment for seduction of a minor. This third pregnancy was realized at the seventh month and the baby shall be given for adoption.

Regarding violence in the women’s history, we observe that it exists in the cases of foreign women that came to Greece seeking a better life. One of the women from Albania stated that she had suffered physical abuse by her husband whom she was pressurized to marry by her parents due to financial reasons; the second from Romania talked about exploitation by her alcoholic grandfather who was her guardian for the period that her mother was living in Greece and, finally, the third from Morocco stated that all her life she had witnessed the beating of her mother by her father, a practice which is acceptable in Moroccan society.

These data confirm studies that show that violence in the family web of the abused pregnant women is a highly frequent phenomenon [56,57] running through generations [9] Exposure to domestic violence during childhood or adolescence can be linked to domestic violence during adult life [57]. The multifactorial nature of family violence has been the scope of international [58] studies that have shown the association of different forms of violence.

The theoretical scientists of social learning support the idea that behavior is shaped by behavior models that the child witnesses in the family of origin [59]. According to this theory, children exposed to domestic violence learn that aggressiveness is an appropriate strategy for managing anxiety and solving conflicts or a way to achieve control both in personal and social relations [60]. Furthermore, children with violent parents may not have the opportunity to learn socially acceptable methods of efficient communication and conflict resolution, such as: negotiation, discussion, self-control tactics and active listening [61].

However, it is worth noting that the family is a protective shield in Greek society given that only two Greek women were found in the women’s shelters. This fact may mean that Greek women abused during pregnancy find protection either in the family or friendly persons to deal with the violent behavior of the partner and, in any case, to avoid the stigma of resorting to a shelter. On the contrary, pregnant women from other places that are, therefore, away from relatives and friends may more easily seek shelter at a public institution.

At this point, we should focus on the profile of these women that came to Greece hoping to find a better life but started going out with people with delinquent behavior; men with alcohol abuse problems and that were aggressive and men that used violence in their family environment as a tool of control and suppression.

We should first of all underline the connection of alcohol use or abuse with violence in pregnancy, which is widely known and supported by various studies [62,63,64]. Often, pregnancy per se is a fact that causes insecurity and presents the future father with responsibilities that he cannot assume, as characteristically explained by the woman from Moldavia. We should notice that the statistical study by the author of this article on domestic violence in Greece showed that foreign pregnant women are in general more affected compared to Greek women. This fact may show that financial factors as well as inclusion factors and the lack of a family support environment are linked to their abuse. Their inability and insecurity, on the one hand, and complete dependence on their partner, on the other, seem to establish ideal conditions for violence by the partner, irrespective of whether he is Greek or foreigner. Therefore, these women, with socioeconomic difficulties wishing to find a partner that would protect them, ended up accepting their violent behavior when he was supposed to assume the role of the father. We underline the fact that based on the women’s statements, violence started during pregnancy in three out of five cases of pregnant women abused by their partner. Developmental psychology believes that people fight unconsciously not only for their personal survival, but also for the continuation of their genetic heritage; this can explain the jealousy, obsessiveness and insecurity of their partners during pregnancy that end up adopting violent behaviors towards their pregnant partners [65].

This fact confirms studies supporting the idea that the factors linked to pregnancy per se may increase the couple’s anxiety and, therefore, the risk of violence. Such factors include unwanted or early pregnancy [66], financial problems [65], and the change in the social role of men and women when they become parents [67].

These findings may be culturally as well as socially and psychologically interpreted. The cultural differences between developed and non-developed countries in matters of violence can represent the differences in percentages relating to violence before and during pregnancy. According to a study, the imbalance degree of the two genders in a society affects the percentages of violence in the general population of women and can also be a factor for violence in pregnancy [26].

In terms of psychology, studies have shown that boys exposed to domestic violence are more likely to become perpetrators as adults, while girls are more likely to become victims as adults compared to children without such family history [68]. In the study of Black et al. [61], most of the participants had witnessed psychological violence in their parents (58.3%). They had also faced psychological violence in their own relationship (69.5%). Similarly, physical violence was observed in the parents (17.5%) who had similar experiences in their relationships with their partners (27.0%).

Cannon et al. [69] found out that 49% of the girls that had witnessed the abusive behavior of their mothers’ partners were the ones that had also suffered domestic violence in childhood.

Finally, regarding the consequences of violence on the physical and mental health of the pregnant woman and the fetus, we observed that in one case, the violence by the former partner resulted in miscarriage, while abortion was the alternative chosen by the other pregnant women as a solution to social exclusion and possible domestic violence that would be unleased. It is worth noting that often women accepted threats against their lives or the life of their children or even suffered violence in front of their children. Anxiety and despair were unquestionably some of the psychological consequences of violence in pregnancy. Regarding physical consequences, it seems that there were none putting the fetus or the reproductive health of the pregnant woman at risk, i.e., hemorrhage or premature rupture of membranes.

Our findings agree with the studies of the World Health Organisation that showed that the consequences of physical violence include bruises, fractures, and broken teeth, while those of psychological abuse are expressed through the development of fears and anxious disorder that can result in substance abuse, post-traumatic stress [70], depression, even suicidal ideation [71,72].

The small number of women included in the collection of qualitative data is a limitation for the research and decreases the reliability index of the results. A qualitative research with a bigger sample and more than one interview could unquestionably give more complete and safer data in the future.

The period of pregnancy and the postpartum period offer opportunities to detect violence and provide women with assistance, as health professionals have access to communication with them [73]. If health professionals are properly trained, they can investigate the possibility of violence using a simple assessment protocol during prenatal care. According to the research carried out on violence prevention and management during pregnancy, it is proven that the best management of violence against pregnant women by her partner is timely detection, as well as the proper training of all involved bodies and the awareness of society in general [74,75,76,77].

This is why, in each prenatal care case at the emergency or regular clinic, health professionals, in particular midwives who openly communicate with the women, must consider the possibility that the pregnant woman is abused. An effort must be made to detect violence; otherwise, the abuse will remain untreated and the mental and physical injuries will continue. Upon confirming the abuse, the follow-up is important to terminate the cycle of violence. The systematic assessment and methodical treatment of each case of violence and the interconnection of welfare and health agencies must be a standard care for all pregnant women [78].

More specifically, according to the quantitative research data, confirmed by the qualitative research, health professionals must be extremely careful in cases of pregnant women with the following characteristics:

- They are immigrants
- They are unemployed and have a low educational level.
- They are single or teenagers.
- Pregnancy is undesirable by the partner.
- They have a history of pregnancy termination (abortion).
- The partner is foreigner.
- There is a great age difference between the women and her partner (i.e., over 10 years)
- They live with their partners but are not married.
- They neglect to have their scheduled tests.
- They already have a child.

Our proposal concerns interventions during pregnancy, as pregnant women will visit the health services at regular intervals for their prenatal care. Health professionals, in particular midwives, must be aware of the above and contribute to the detection of such cases of violence and their effective treatment.

### Limitations of Phenomenological Methodology

Unquestionably, the phenomenological methodology is subject to the limitations of the qualitative research which is based on an interview. Some of the problems that arise, due to bias, are, for example, the willingness of the subject to give the answers the researcher wants or the answers that shall be socially acceptable and desirable, or even the tendency of the researcher to seek answers already on his/her mind. It is a “subtle competition occurring sometimes between the researcher and the respondent [79].

The personal opinions of the subjects cannot be controlled, as there is this feeling during the interview that there is a kind of pressure on the subject to “say something significant”. The respondents deep down nurture the hope that they shall give the “proper” answers. But this is not the desirable conclusion of an interview. The aim of the researcher is to approach and record the truth, without processing and discoloring the answers. The subjects change their answers from the moment they realize, by the researcher’s reaction, that they have contributed to the expected outcomes of the research.

Although the answers of the respondents must not be encouraged or directed by the researcher’s questions, the latter often uses certain approaches, including the encouragement of the subject or patronizing or interest. Empathy can also be used, when the subject starts narrating a serious personal topic which is indifferent for the research but the respondent wishes to share it with the researcher. More specifically, though, in terms of personal issues, the questions must change or be posed in such a way so as not to harm the subject’s psyche.

With regard to this research, the circumstances that the abuse started for the specific sample are studied and the profiling of the abused woman is attempted. Finally, the dimension of the impacts brought on by the abuse to the reproductive health of the pregnant woman and the fetus are investigated.

## 5. Conclusions

The aim of this research was to study in depth, as much as possible, the experiences of pregnant women who are abused and bring to light the factors associated with the abuse and the consequences for women and fetuses. Thus, the semi-structured interview was used to investigate the way the experience of violence is recorded by the pregnant women, using the phenomenological approach. The results show that domestic violence against pregnant women is significantly correlated to the poor socioeconomic and educational background of the women and their partners. These were mainly the cases of immigrant women searching for a better life in Greece. On the other hand, Greek women who have experienced violence during pregnancy were severely traumatized during their lives. This result demonstrates that women who resort to shelters because of abuse during pregnancy are women for whom violence was almost a routine in their lives. On the other hand, perpetrators are partners or family members addicted to drugs or alcohol and have a low socioeconomic status. As abortion and miscarriages are possible outcomes of the violence experienced and the psychological impact on the mother and the fetus can have severe consequences in their future lives, it would be wise to investigate in depth and on a larger scale the phenomenon of violence during pregnancy, so as to prevent its perpetuation. Social and health policy strategies are important for the treatment of the problem, both for Greek women and immigrants. The following are included in this context: (a) prenatal care and reproductive health polices; (b) information, awareness and networking actions: (c) training of public administration employees for the proper and more in-depth treatment in cases of violence against women; (d) networking of the municipal-based centers for the prevention and treatment of the phenomena of violence; (e) setting up consulting centers and their staffing with trained staff to be able to provide pregnant women with assistance as well as the necessary psychological and social support. The aim is to allow woman to be able to make the best decisions for her future and the future of her child. The above-analyzed public policy proposals form a social model promoting respect for human rights and is a guarantee that there are founded expectations for these societies that they will be able to effectively deal with the phenomenon of violence, 1which has extremely adverse effects, in particular during pregnancy.

## Figures and Tables

**Table 1:** Profile of abused pregnant woman.

Time of stay at	
the Guesthouse for abused women of the National Social Solidarity Center	4
Mitera	3
Demographic characteristics	
Nationality: Greek	2
Foreign	5
Illegal immigration status	4
Age (years): 18–25	4
26–35	2
36–40	1
Age of first pregnancy	
<18	3
>18	4
Education	
Primary education	2
Secondary education	3
Higher education	1
Work status	
Unemployed	4
Occasional—Not insured work	2
Private employee	1
Marital status	
Married	3
Single (living with a partner)	2
Single (living alone)	2
Number of pregnant women with living children	4 (1 woman had given her babies for adoption)
Miscarriages due to abuse	3
Abortions	3

results according to the answers of the women during the interview.

**Table 2:** Characteristics of violence.

Start of violence: before pregnancy (continued in pregnancy as well)	Giota, Lambrini, Susanna, Ariadne,
During pregnancy	Elena, Lena, Lambrini, Marina
Type of violence: physical (kicking, slapping on the face, using objects to beat, pushing)	Giota, Elena, Lena, Marina, Ariadne, Susanna
Psychological (threats, bulling)	Giota, Lena, Lambrini, Marina, Susanna, Ariadne
Sexual	Lena, Lambrini, Marina, Ariadne
Perpetrator: partner/husband	5

results according to the answers of the women during the interview.

**Table 3:** Partner’s/husband’s profile.

Start of Violence: Before Pregnancy (Continued in Pregnancy as Well)	Giota, Lambrini, Susanna, Ariadne,
During Pregnancy	Elena, Lena, Lambrini, Marina
Type of violence: physical (kicking, slapping on the face, using objects to beat, pushing)	Giota, Elena, Lena, Marina, Ariadne, Susanna
Psychological (threats, bulling)	Giota, Lena, Lambrini, Marina, Susanna, Ariadne
Sexual	Lena, Lambrini, Marina, Ariadne
Perpetrator: partner/husband	5
Father/other family member	2

results according to the answers of the women during the interview.
